# Recent Advances and Unaddressed Challenges in Biomimetic Olfactory- and Taste-Based Biosensors: Moving Towards Integrated, AI-Powered, and Market-Ready Sensing Systems

**DOI:** 10.3390/s25227000

**Published:** 2025-11-16

**Authors:** Zunaira Khalid, Yuqi Chen, Xinyi Liu, Beenish Noureen, Yating Chen, Miaomiao Wang, Yao Ma, Liping Du, Chunsheng Wu

**Affiliations:** 1 Institute of Medical Engineering, Department of Biophysics, School of Basic Medical Sciences, Health Science Center, Xi’an Jiaotong University, Xi’an 710061, China; zunairakhalid@stu.xjtu.edu.cn (Z.K.); chen-yuqi@stu.xjtu.edu.cn (Y.C.); xinyiliu599@stu.xjtu.edu.cn (X.L.); beenishktk@stu.xjtu.edu.cn (B.N.); ytc20201011@stu.xjtu.edu.cn (Y.C.); wmm15029418463@stu.xjtu.edu.cn (M.W.); yaoma@stu.xjtu.edu.cn (Y.M.); 2 Key Laboratory of Environment and Genes Related to Diseases (Xi’an Jiaotong University), Ministry of Education of China, Xi’an 710061, China

**Keywords:** biomimetic biosensors, olfactory–taste integration, hybrid sensing platforms, artificial intelligence (AI), microfluidics, nanomaterials

## Abstract

**Highlights:**

**What are the main findings?**

- Hybrid biomimetic olfactory–taste systems, strengthened by advanced recognition elements and nanomaterials, outperform single-modality sensors in sensitivity, selectivity, and robustness.
- AI-driven analytics (e.g., drift compensation, data fusion, and forecasting) make these platforms more reliable and predictive on real-world samples.

**What are the implications of the main findings?**

- Integrated “e-panel” sensors (olfaction + taste) enable on-site, real-time decisions for food safety, healthcare, and environmental monitoring.
- The convergence of biomimetic interfaces, modern materials, and AI is accelerating translation toward practical, market-ready applications.
- Recent progress in olfactory and taste biosensors has been driven by advances in nanomaterials, recognition elements, and AI-based data processing.
- Hybrid systems that combine olfactory and taste sensing outperform single-modality sensors, offering higher sensitivity, selectivity, and robustness.
- Integrating olfactory and taste functions into one sensor platform opens new opportunities for food safety, healthcare, and environmental monitoring.
- These hybrid, AI-enabled biosensors are moving closer to practical, market-ready applications.

**Abstract:**

Biomimetic olfactory and taste biosensors replicate human sensory functions by coupling selective biological recognition elements (such as receptors, binding proteins, or synthetic mimics) with highly sensitive transducers (including electrochemical, transistor, optical, and mechanical types). This review summarizes recent progress in olfactory and taste biosensors focusing on three key areas: (i) materials and device design, (ii) artificial intelligence (AI) and data fusion for real-time decision-making, and (iii) pathways for practical application, including hybrid platforms, Internet of Things (IoT) connectivity, and regulatory considerations. We provide a comparative analysis of smell and taste sensing methods, emphasizing cases where integrating both modalities enhances sensitivity, selectivity, detection limits, and reliability in complex environments like food, environmental monitoring, healthcare, and security. Ongoing challenges are addressed with emerging solutions such as antifouling/self-healing interfaces, modular cartridges, machine learning (ML)-assisted calibration, and manufacturing-friendly approaches using scalable microfabrication and sustainable materials. The review concludes with a practical roadmap advocating for the joint development of receptors, materials, and algorithms; establishment of open standards for long-term stability; implementation of explainable/edge AI with privacy-focused analytics; and proactive collaboration with regulatory bodies. Collectively, these strategies aim to advance biomimetic smell and taste biosensors from experimental prototypes to dependable, commercially viable tools for continuous chemical sensing in real-world applications.

## 1. Introduction

Biomimetic olfactory and taste biosensors are directly inspired by the human senses of smell and taste. They use biorecognition molecules, bioengineered receptors, and transducer components to mimic how our bodies detect and interpret chemical signals [1,2]. By selectively binding to target molecules and converting these interactions into measurable signals, these sensors achieve high sensitivity and specificity, which is essential for critical applications such as food safety, environmental monitoring, security screening, and medical diagnostics [3,4]. Some designs incorporate advanced biological elements such as organoids or the ligand-binding domains of human taste receptors to emulate natural sensing pathways, enabling detection at picomolar concentrations and the ability to distinguish complex analyte mixtures, even in challenging real-world samples [5]. In addition to biological components, new materials enhance the durability and versatility of these sensors. Antifouling films, molecularly imprinted layers, and nanostructures such as graphene, metal–organic frameworks (MOFs), and gold nanoparticles improve the devices’ stability and performance across food, environmental, and health fields [6,7,8].

Beyond biomimetic, receptor-based platforms, conventional electrochemical and metal-oxide–semiconductor (MOS) gas sensors—often deployed as e-nose/e-tongue arrays—are widely used for related tasks in food and environmental monitoring. In practice, MOS and conducting-polymer arrays analyzed with pattern-recognition (e.g., PCA pipelines) have been applied to odorant/flavor VOCs with an emphasis on portability and AI integration [1]. These conventional approaches, while cost-effective, typically trade molecular selectivity for pattern-based discrimination and can face issues such as poor selectivity and slow regeneration under variable humidity, necessitating calibration/drift management [9]. Classic electronic-tongue systems are also established in the food industry for quantifying basic tastes, underscoring complementarity with receptor-targeted biomimetic strategies surveyed here [10].

Recent food-focused work in 2025 shows e-noses/e-tongues moving from static lab classifiers to predictive, on-site quality control. For freshness and shelf-life, multi-step forecasting of beef total viable count from e-nose time-series achieved strong predictive fits across 1–9 h horizons [11], slaughterhouse inspection reported high sensitivity/specificity for contamination and aging classes [12], and seafood workflows paired portable arrays with statistical process control to trigger real-time alarms aligned with microbiology [13]. For authenticity and fraud, a portable MOS e-nose separated EVOO/VOO/LOO and quantified sunflower-oil adulteration with robust PLS performance [14]; oolong teas were classified by geographical origin at near-ceiling accuracy with sensor-importance analysis [15]; and hybrid GC–e-nose pipelines discriminated roasted coffees across multiple origins [16]. In beverages, handheld systems detected cork taint (TCA ≥ 2 ng/L) with high accuracy and supported quantitative models [17]. In dairy safety, e-nose + ML enabled rapid, non-destructive melamine screening in milk powders [18]. (Consumer-facing profiling also reached 92–100% cross-validated accuracy on coffee capsule “sensory classes” using an e-tongue vs. e-noses benchmark [19]).

Recent advancements in materials science, device design, and artificial intelligence have significantly enhanced the sensitivity, selectivity, and operational stability of biomimetic olfactory and taste biosensors. Hardware developments have also advance rapidly. Organic electrochemical transistors (OECTs) that mimic synapses, chemi-memristive gas sensors, and organic field-effect transistor (OFET) arrays equipped with in-sensor reservoir computing now achieve extremely low detection limits, offer fast responses, and can capture complex time-dependent patterns [20,21,22]. Nanotechnology remains pivotal, with innovations such as olfactory receptors, carbon nanotubes, enzymatic labels, and nucleic-acid amplification techniques boosting signal strength and reducing noise [23]. The incorporation of AI—utilizing machine learning, deep learning, and statistical pattern recognition algorithms—has transformed data processing, enabling sensors to classify complex chemical mixtures, detect subtle changes in analyte profiles, and adapt to varying environmental conditions [3,24,25]. Moreover, the convergence of AI-powered biosensing with Internet of Things (IoTs) platforms and wearable device is expanding real-time, on-site diagnostic capabilities, paving the way for more autonomous and context-aware sensing systems that effectively bridge the gap between laboratory research and practical field applications [2,26].

To contextualize biomimetic platforms against widely used non-bio gas sensors, we provide a side-by-side comparison of operating conditions (e.g., selectivity, LOD, drift, power, and maintenance) in Table 1.

Although significant progress has been made in biomimetic olfactory- and taste-based biosensors, several major challenges remain. Most current reviews treat these sensing methods separately, missing opportunities for cross-platform comparisons, shared design strategies, and combined hybrid approaches [1]. While artificial intelligence—especially in complex pattern recognition and real-time decision-making—is recognized, it is often viewed as an optional addition rather than a core element integrated with advances in material and device design. Furthermore, critical issues such as sensor stability, reproducibility under varying environmental conditions, and long-term operational reliability receive limited focus, which hampers the transition from promising lab results into consistent performance in practical applications. The pathways to commercialization are also insufficiently examined, with minimal discussion of regulatory challenges, manufacturing scalability, or cost-effectiveness, all critical factors for moving beyond prototype stages. Collectively, these shortcomings highlight the need for a comprehensive, multidisciplinary framework that integrates materials science, AI-powered analytics, and engineering design to overcome both technical and translational obstacles.

To bridge these gaps, this review adopts a multidisciplinary approach, exploring how advances in sensing materials, AI-powered data processing, and system-level engineering can mutually enhance one another to overcome ongoing challenges. Rather than treating these developments separately, the focus is on their interconnectedness—for example, how innovations in materials can boost AI feature extraction, or how device design decision in devices influence the accuracy and stability of algorithms [3]. By directly comparing olfactory and taste biosensors and exploring hybrid configurations, the review aims to reveal synergies that could accelerate the transition from experimental prototypes to reliable, market-ready solutions in areas such as food safety, healthcare, and environmental monitoring.

This review, focusing on recent studies, covers three main themes: innovations in materials and devices, AI-powered performance enhancements, and strategies for practical application. It considers both olfactory and taste sensing simultaneously, paying special attention to combined platforms when applicable. The structure of the review is as follows: Section 3 outlines the biological principles that motivate biomimetic olfactory and taste sensing; Section 4 surveys advances in recognition materials, signal transduction, and device development; Section 5 integrates AI and data-fusion approaches for robust inference; Section 6 examines hybrid (olfactory and taste) and multiplexed architectures; Section 7 discusses translation and commercialization pathways; and Section 8 highlights future research directions. Our work offers a multidisciplinary overview connecting materials and device design with learning algorithms and real-world implementation, emphasizing comprehensive co-design and evaluation metrics.

We make the biology → engineering link explicit via a five-step pathway used throughout this review: **mechanism** (ligand–receptor binding, combinatorial coding) → **design rules** (affinity/kinetics, hydration/ionic milieu, noise control, array diversity) → **interfaces** (recombinant ORs/OBPs, peptides/aptamers or MIPs on oriented linkers and antifouling coatings, lipid/hydrogel supports) → **transduction** matched to time constants (FET/OECT, impedance/electrochemical/MEAs, optical) → **data/AI** (baseline correction/drift handling; classical ML to modern deep models). Collectively, the subsequent sections outline a practical pathway—from biological recognition principles (Section 3), through materials and device engineering (Section 4), to AI, data fusion, and real-time analytics/IoT (Section 5), into hybrid and multiplexed systems for cross-modal sensing (Section 6), and finally to translation, market pathways, and regulatory/manufacturing alignment (Section 7).

## 2. Methodology for Literature Collection and Analysis

We conducted a structured literature search to identify recent primary studies and reviews on biomimetic olfactory and taste biosensors, hybrid (e-nose/e-tongue) systems, and AI-enabled sensing workflows. Searches were run in Web of Science, Scopus, PubMed, and IEEE Xplore, with supplementary snowballing via Google Scholar. The core time window was January 2015–October 2025 to capture modern transducer, nanomaterials, and AI developments; a limited number of pre-2015 publications were included when they provided foundational concepts (e.g., early OBP/FET work, classic e-tongue texts) that remain relevant. We combined controlled vocabulary and keywords; representative Boolean strings included: (“bioelectronic nose” OR “biomimetic olfactor” OR “olfactory receptor” OR “odorant-binding protein” OR “OR-FET” OR “graphene FET” OR “OECT”) AND (sensor OR biosensor) and (“electronic tongue” OR “taste receptor” OR “T1R” OR “T2R” OR “TRPV1” OR “chemical tongue”) AND (biosensor OR sensor), together with (“multimodal” OR “hybrid” OR “fusion”) AND (nose OR tongue) and (machine learning OR deep learning OR domain adaptation OR data fusion). Inclusion criteria were: peer-reviewed articles (and a small number of book chapters where they provide methods or device fundamentals), English language, clear experimental or engineering content on biomimetic recognition (receptors, OBPs, peptides, MIPs, organoids/cells) or established e-nose/e-tongue arrays used as comparators, and quantitative performance/metrics when available. Exclusion criteria were: patents, theses, preprints without peer review, purely computational works with no sensing interface, studies focused solely on non-bio chemi-resistors unless used as comparators, non-English articles, and items with retraction/major integrity flags. Records were deduplicated across databases, then screened by title/abstract; full texts were reviewed for eligibility and to extract device class, recognition element, transducer, sample domain, and performance metrics. Snowballing from reference lists added recent or seminal items missed by indexing. The resulting corpus underpins the tables and comparisons in this review; pre-2015 citations are retained selectively for foundational context, while the emphasis of the analysis is on 2015–2025 advances. Where applicable, we note grey-area cases (e.g., conventional MOS arrays) explicitly as comparators rather than biomimetic exemplars to maintain a clear scope. The distribution of publisher domains for the included studies (n = 147) is summarized in Table 2 (MDPI 38.10%, Elsevier/ScienceDirect 25.85%, ACS 8.84%, Wiley 6.12%, Nature/Springer Nature 4.76%, SpringerOpen/BMC 2.04%, Frontiers 2.72%, RSC 1.36%, IEEE Xplore 1.36%, ECS Digital Library 1.36%, AAAS 1.36%, Others 6.12%).

## 3. Principles of Olfactory and Taste Biosensors

### 3.1. Biological Basis

The mammalian olfactory and gustatory systems serve as highly specialized chemical detection networks, capable of recognizing and discriminating complex stimuli even at trace concentrations. In the olfactory system, detection begins in the main olfactory epithelium, where each olfactory sensory neurons (OSNs) expresses a single odorant receptor (OR), which belongs to the G protein-coupled receptor (GPCR) family [39]. Axons from OSNs that express the same OR converge onto specific glomeruli in the olfactory bulb, forming a chemospatial map that preserves receptor identity and supports odor discrimination [40,41]. This precise organization, maintained despite continuous OSN renewal, enables the system to detect volatile compounds at concentrations below the picomolar range. Signals from glomeruli are processed by mitral and tufted cells and relayed to higher brain areas, including the piriform cortex, where both innate and learned odor recognition take place [42]. Accessory olfactory structures, such as the vomeronasal organ, contribute to pheromone detection and social communication, with their functions integrated at the amygdala level.

The gustatory system detects non-volatile chemical stimuli through specialized taste receptor cells located in taste buds on the tongue and within the oral cavity. There are five canonical taste modalities—sweet, umami, bitter, salty, and sour—each mediated by distinct receptor mechanisms [43]. Sweet and umami tastes are detected by heterodimers of T1R GPCRs, specifically T1R2/T1R3 for sweet and T1R1/T1R3 for umami, while bitter taste relies on the T2R GPCR family, which includes approximately 25–26 functional variants in humans [44]. Salty and sour tastes are mediated by ion channels. Beyond the oral cavity, T1R and T2R receptors are also presented in the gastrointestinal tract and other tissues, where they participate in nutrient sensing, metabolic regulation, and immune functions, such as detecting bacterial quorum-sensing molecules [45].

**Figure 1:**
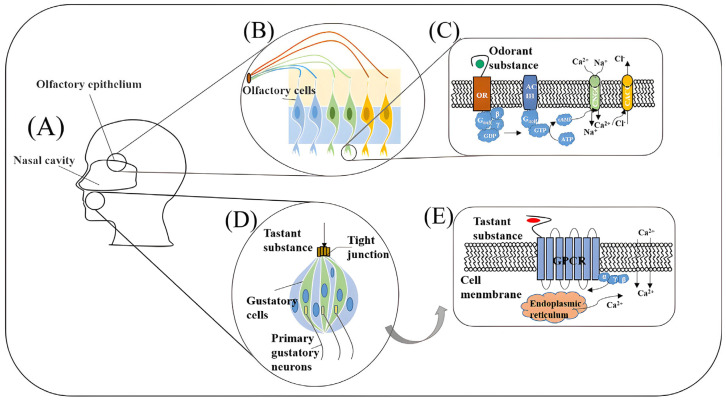
Biological olfactory and gustatory pathways and their biosensor analogues (**A**) Sites of olfactory epithelium and taste buds; (**B**) olfactory epithelium with receptor-specific neurons converging to glomeruli (sensor-array analogue); (**C**) odorant–OR GPCR cascade → ACIII → cAMP → CNG channels → Na^+^/Ca^2+^ influx and Cl^−^ efflux; (**D**) taste bud with tight-junctioned taste receptor cells contacting primary afferents; (**E**) tastant–GPCR (T1R/T2R) signaling via PLCβ_2_–IP_3_ → Ca^2+^ release/TRPM5 activation [1].

Signal transduction in both olfactory and gustatory systems involve the binding of ligand-receptor, activation of G proteins, and initiation of intracellular signaling pathways like the IP3/Ca^2+^ pathway, which ultimately leads to membrane depolarization and neurotransmitter release. While both systems rely on GPCR-mediated detection for many modalities, olfaction primarily processes a wide combinatorial array of volatile molecules, whereas gustation is tuned to a narrower, modality-specific chemical space. These shared and distinct features directly guide design choices: GPCR-mediated binding and interfacial charge redistribution favor FET/OECT or impedance readouts; ion-channel/Ca^2+^ signaling aligns with impedance or optical transducers; and spiking/combinatorial coding motivate arrayed sensors with differential/reference channels and ML-based decoding. Figure 1 illustrates the natural olfactory and gustatory systems alongside their biomimetic biosensor counterparts, highlighting similarities in recognition mechanisms, signal transduction methods, and data processing workflows.

### 3.2. Signal Transduction Mechanisms

The conversion of biochemical recognition events into measurable signals is a fundamental process in biomimetic olfactory and gustatory biosensors, enabling the detection of volatile and non-volatile analytes with high sensitivity and specificity. Various transduction methods have been developed to convert molecular interactions at the biorecognition interface into measurable electrical, optical, or mechanical signals.

Electrochemical transduction remains the most widely adopted approach, leveraging techniques such as cyclic voltammetry, differential pulse voltammetry, and electrochemical impedance spectroscopy to monitor redox reactions or impedance changes induced by ligand–receptor binding [46]. Field-effect transistor (FET)-based platforms, including low-dimensional FETs and organic electrochemical transistors (OECTs), provide inherent signal amplification and rapid responses. Incorporating nanomaterial such as graphene and transition metal dichalcogenides (TMDCs) further enhancing electron mobility, sensitivity, and stability [47]. Microelectrode array (MEA) technology enables direct recording of extracellular potentials from taste or olfactory receptor cells, allowing for the correlation of specific spiking patterns with analyte identity [48].

Optical transduction methods, including surface plasmon resonance (SPR), fluorescence-based calcium imaging, interferometric reflectance, and metasurface-enhanced sensing, offer label-free and real-time detection by monitoring refractive index changes, luminescence, or light–matter interaction shifts upon analyte binding [49]. These approaches are particularly advantageous for multiplexed biosensing, although their performance can be influenced by environmental optical noise.

Mechanical transduction exploits piezoelectric and quartz crystal microbalance (QCM) systems to detect mass loading on sensor surfaces, converting molecular adsorption at the nanogram-scale into frequency shifts. Integration with nanostructured coatings or biomimetic recognition layers improves selectivity and minimizes interference, enabling reliable detection in complex samples [50].

To extend detection limits and improve selectivity, signal amplification strategies are embedded within transduction architectures. Nanomaterials such as gold nanoparticles, carbon nanotubes, and nanozymes enhance electrode surface area and catalytic activity, while nucleic acid amplification reactions (e.g., hybrid chain reaction) provide enzyme-free, high-gain signal enhancement. Immobilized multi-enzyme or nanozyme cascades mimic natural catalytic pathways, delivering stable and synergistic amplification for continuous monitoring [51].

Interface engineering further enhances transduction efficiency. Well-designed surfaces such as molecularly imprinted polymers (MIPs), antifouling coatings, and S-layer protein films—help properly orient receptors, reduce non-specific binding, and preserve sensitivity even in complex, real-world fluids [52]. These advancements enable biosensors to perform reliably in physiologically relevant environments, which is essential for practical applications.

**Figure 2:**
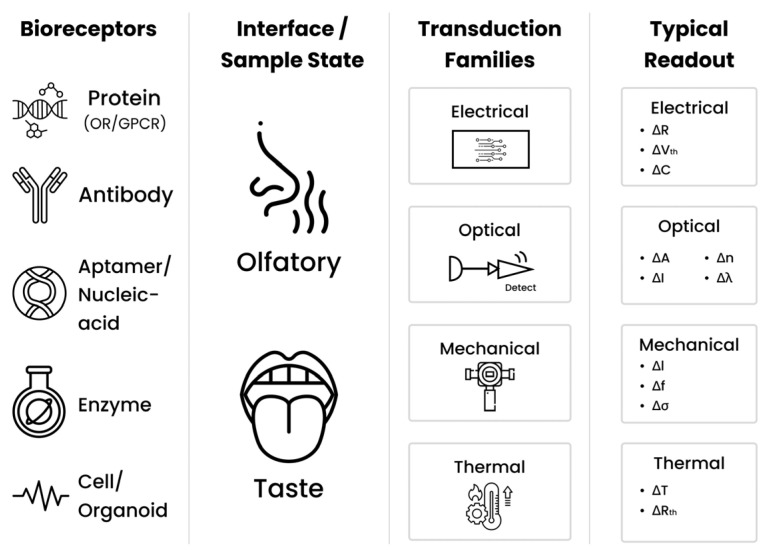
Principal transduction strategies in biomimetic olfactory and taste biosensors.

Overall, the diverse transduction methods, including electrochemical, optical, mechanical, and transistor-based approaches, provide these biosensors with the flexibility required for applications in food safety, environmental monitoring, and medical diagnostics. Advances in incorporating nanomaterials, enhancing amplification chemistries, and modifying surface and interface has significantly improved their sensitivity, stability, and ability to detect multiple targets simultaneously, moving bioinspired sensing technologies toward practical and scalable implementations. A schematic diagram summarizing the principal transduction strategies employed in biomimetic olfactory and taste biosensors is shown in Figure 2.

This three-panel layout separates **classification**, **signal conversion**, and **selection rationale**, reducing ambiguity and providing a quick pathway from Section 3.2 concepts to practical design choices.

### 3.3. Performance Metrics

These sensors are primarily evaluated based on sensitivity, selectivity, and limit of detection (LOD), as well as their response and recovery speeds, and ability to perform in challenging conditions.

Sensitivity reflects the biosensor’s ability to detect very low concentrations of target analytes and is often enhanced via nanomaterial-based transduction layers (e.g., metal nanoparticles, graphene, quantum dots) and optimized immobilization of biorecognition elements on transducer surfaces [1,46]. Signal amplification approaches, including enzymatic catalysis, nucleic acid amplification, and conductive polymers, further enhance detection limits to sub-nanomolar or even parts-per-billion (ppb) levels.

Selectivity describes the biosensor’s ability to distinguish target analytes from structurally similar or interfering compounds. This is improved through the use of odor-binding proteins, peptides, and functionalized nanomaterials for better molecular recognition, alongside pattern-recognition algorithms that analyze complex mixtures [53]. The LOD represents the smallest concentration that can be reliably detected, which depends on factors like noise reduction and transducer sensitivity; novel metrics like effective sensitivity integrate maximum and average responses to better reflect real-world variability [54].

Response and recovery times are critical for real-time applications, with high-performance systems achieving cycle times from sub-second to few seconds. Stability and reproducibility are assessed through long-term operational tests under variable humidity, temperature, and contaminant exposure. Emerging sensor designs also incorporate neuromorphic features, such as short- and long-term memory functions, to improve adaptability in changing environments [21]. Collectively, these metrics provide a quantitative framework for comparing devices and guiding the advancement of next-generation bioinspired sensing technologies.

## 4. Advances in Materials & Device Engineering

The advancement of biomimetic olfactory and taste biosensors has been driven by simultaneous progress in materials science, nanotechnology, biochemical engineering, and device integration. These breakthroughs are central to improving core performance metrics such as sensitivity, selectivity, stability, and detection limits. Over the past few years, the field has shifted from isolated incremental improvements to adopting comprehensive engineering approaches—combining natural and synthetic recognition elements, nanomaterial-based and biochemical amplification methods, and next-generation transducer systems integrated with microfluidics and portable platforms.

Importantly, comparative analyses of olfactory and taste systems reveal unique material-transducer combinations tailored to the volatility, size, and binding kinetics of their respective targets, as well as potential for cross-platform integration. This section consolidates recent progress across these domains, culminating in a discussion of challenges that persist and the emerging solutions designed to overcome them.

### 4.1. Recognition Elements & Biorecognition Strategies

The evolution of recognition elements in biomimetic olfactory and taste biosensors has been marked by parallel progress in both natural bioreceptors and synthetic or engineered materials, each tackling essential challenges related to sensitivity, selectivity, and operational stability. Natural recognition elements—such as olfactory and taste receptors, odorant-binding proteins, and cell-based sensing units—offer unmatched biological specificity and compatibility, enabling precise detection of volatile and non-volatile compounds. Advances in immobilization strategies, including oriented attachment, chemical linker chemistries, and extracellular matrix-based supports, have enhanced their structural stability and extended their functional lifespan [1].

In parallel, synthetic receptors have been developed to overcome limitations of natural receptors, including fragility, expense, and inconsistency, while maintaining strong binding affinity. Molecularly imprinted polymers (MIPs) have gained prominence due to their high affinity, robustness, and cost-effectiveness, with recent studies integrating MIPs into electrochemical and optical sensor systems to achieve selective, reproducible, and field-deployable detection [55]. Other synthetic formats—such as aptamers, affibodies, and engineered peptides—provide adjustable specificity and enhance stability across various environmental conditions [56]. Hybrid approaches, such as MIP–aptamer composites, combine the durability of polymers with the molecular recognition abilities of nucleic acids, enabling ultra-low detection limits.

Emerging recognition strategies also utilize bioinspired synthetic systems like the “chemical tongue”, which employs polymeric and polyamino acid-based materials functionalized with environment-responsive fluorophores to mimic gustatory discrimination across complex mixtures. Coupled with statistical and machine-learning algorithms, these systems offer high-throughput, label-free analysis of biological samples, expanding applicability to clinical diagnostics and environmental monitoring [57]. In the olfactory domain, biohybrid systems integrating nanomaterial-enhanced electronic noses with receptor-based sensing arrays have improved selectivity and signal-to-noise ratios for detecting volatile organic compound, benefiting applications in food safety, environmental monitoring, and medical breath analysis [3].

Table 3 and Table 4 summarizes recent olfactory- and taste-based biosensors, respectively, combining recognition elements with FET and electrochemical transducers, detection limits, selectivity, and applications. This integrated view highlights how receptor chemistry and device design together enable ultra-low detection and sets the stage for amplification strategies. The integration of recognition chemistry with nanoscale transducers forms the foundation of high-performance, field-ready biosensing platforms [58].

### 4.2. Signal Amplification & Sensitivity Enhancement

Recent advances in nanomaterial-based and biochemical amplification techniques have markedly enhanced the sensitivity, selectivity, and detection limits of biomimetic olfactory and taste biosensors. Nanomaterials such as gold and silver nanoparticles, carbon nanotubes, graphene derivatives, quantum dots, and metal–organic frameworks offer large surface-to-volume ratios, high conductivity, and unique catalytic or optical features that enhance signal transduction and reduce detection thresholds [81]. For example, plasmonic nanoparticles amplify optical signals, while carbon nanomaterials improve electron transfer and enable rapid responses.

Biochemical amplification approaches—such as enzymatic labelling, nucleic acid amplification (e.g., PCR, strand displacement, and rolling circle amplification), and hybridization chain reactions—further boost sensitivity by exponentially amplifying target recognition events [82]. Enzymes offer high catalytic efficiency and specificity, whereas nucleic acid amplification achieves outstanding detection limits for low-abundance analytes.

Hybrid amplification systems that integrate nanomaterials with biomolecular recognition elements have emerged as a particularly effective strategy. Nanobioconjugates—combining nanoparticles, quantum dots, or magnetic nanostructures with proteins, aptamers, or DNA/RNA—achieve synergistic effects by coupling high surface area and tailored electronic properties with molecular specificity [83,84]. These hybrid platforms have demonstrated multiple-fold improvements in sensitivity, broader dynamic ranges, and improved selectivity, even when analysing complex samples.

**Figure 3:**
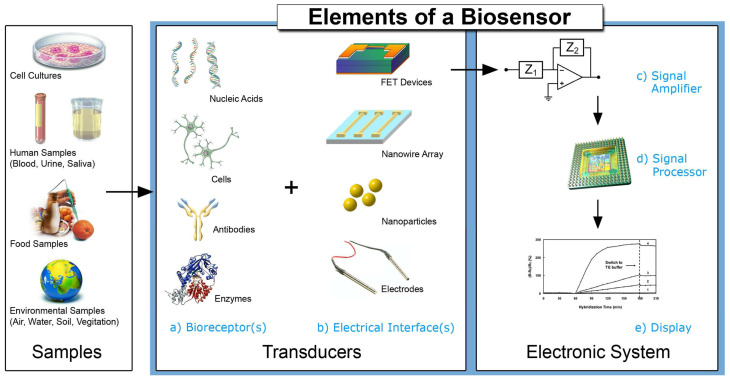
Recognition elements and amplification strategies integrated with nanomaterial-based transducers in biomimetic biosensors [58].

Innovations in composite and hierarchical nanostructures have also contributed to improved mechanical stability, biocompatibility, and operational robustness, enabling integration into miniaturized and portable biosensor devices [85]. Together, these amplification strategies are accelerating the transition of olfactory and taste biosensors from laboratory prototypes toward reliable, real-world-ready analytical systems. The generic biosensor architecture—linking sample types to (a) biorecognition elements and (b) electrical interfaces, and then to the electronic chain ((c) signal amplifier, (d) processor, (e) display)—is summarized in Figure 3.

### 4.3. Transducer Technologies & Device Integration

Recent advances in transducer technologies and device integration have significantly enhanced the functionality, sensitivity, and scalability of biomimetic olfactory and taste biosensors. Electrochemical platforms such as field-effect transistors (FETs), ion-sensitive FETs (ISFETs), and electrochemical impedance spectroscopy (EIS) systems have emerged as key enablers, offering high sensitivity, low detection limits, and real-time monitoring capabilities [1]. Optical transducers, including surface plasmon resonance (SPR), fluorescence, and interferometry-based techniques, provide label-free detection with excellent specificity, while mechanical platforms such as quartz crystal microbalance (QCM) and microcantilever systems enable precise measurements of mass changes [86].

To enable cross-modal (e-nose + e-tongue) systems, device choices must be integration-compatible: gas vs. liquid sampling paths, hydration/ionic requirements of bio-interfaces, and shared operating windows (pH, temperature) should be co-designed. On the data side, successful fusion requires measurements from the same sample/timepoint, followed by pre-processing/normalization and block scaling before low-, mid-, or high-level fusion; otherwise, the largest/noisiest block can dominate and bias models [87]. In high-ionic media, nano-FET/ISFET readout is limited by Debye screening; OECTs mitigate this via low-voltage volumetric gating in aqueous electrolytes, while practical FET mitigations include nanostructured interfaces, shorter receptors/aptamers, and non-equilibrium readouts [88]. Electrochemical/impedance channels are natively liquid-compatible and pair with reference electrodes, but hybrid nose–tongue platforms need coordinated headspace vs. liquid sampling and ionic-strength alignment across channels to limit cross-talk and calibration drift [89]. Optical modes integrate best when optical alignment and refractive-index/temperature stability are controlled; in fusion, their high feature dimensionality often favors mid-level fusion (feature selection or latent-variable models) over raw concatenation [90]. For mechanical/QCM co-integration, humidity control, viscoelastic loading in liquids, and flow-path isolation are required to avoid cross-talk with gas-phase modules prior to fusion [91].

The integration with microfluidics has been particularly transformative, allowing for precise fluid handling, reduced sample consumption, and automation, which supports high-throughput assays and organ-on-chip models for simulating complex sensory environments [1,92]. Miniaturizing these systems makes it portable and suitable for field use, paving the way for point-of-care tests, on-site environmental monitoring, and rapid food-safety assessments.

Wearable and portable devices, often incorporating flexible substrates and equipped with wireless modules, have emerged as the next-generation of real-time detection platforms. Advanced designs utilize multi-channel carbon nanotube FET (CNT-FET) arrays or optical microelectrode arrays to enable multiplexed analyte detection with high selectivity [67]. Furthermore, the integration of biological components such as taste and olfactory receptors, either through in vitro cell- or tissue-based approaches or in vivo implantable microelectrodes, continues to enhance selectivity and mimicry of natural sensory pathways [86]. Because fusion can amplify channel-to-channel drift/aging, practical deployments pair fusion with calibration transfer/standardization and domain-adaptation models to maintain accuracy over time [93].

For cross-modal (e-nose + e-tongue) systems, co-design should ensure: (i) shared operating windows (pH/temperature; hydration/ionic strength) so biointerfaces remain active; (ii) sampling alignment (synchronized headspace vs. liquid routes; same sample/timepoint) before fusion; (iii) signal compatibility—FET/OECT when interfacial charge in electrolytes is dominant (OECTs tolerate high-ionic media and low-voltage operation; nano-FETs require mitigation of Debye screening via interface design or dynamic readouts), impedance/optical for ion/Ca^2+^ flux, and MEA for spiking; and (iv) packaging & cross-talk control (flow-path isolation, shielding, reference/differential channels). Block scaling/normalization prior to fusion prevents any single data block from dominating multichannel models [94].

### 4.4. Comparative Analysis of Olfactory vs. Taste Biosensor Engineering

While biomimetic olfactory and taste biosensors share fundamental design principles, but their materials and device engineering approaches differ to meet the unique demands of each sense. Olfactory biosensors commonly employ nanomaterial-based signal amplification, incorporating metal nanoparticles, carbon nanotubes, and quantum dots to enhance sensitivity and enable detection of complex volatile organic compounds. In contrast, taste biosensors often integrate enzyme-based amplification, nucleic acid amplification methods, or electrochemical and optical transducers optimized for identifying dissolved taste-active compounds [1].

Microfluidic systems and organ-on-chip models have emerged as versatile tools applicable to both types, enabling high-throughput assays, precise fluid control, and the simulation of complex biological interactions relevant to both modalities [92]. Additionally, the use of organoids and hybrid material architectures offers biological realism, improving selectivity, stability, and reproducibility across these platforms. Olfactory modules emphasize volatile capture (pre-concentration, humidity/temperature control) and larger arrays for combinatorial coding, whereas taste modules emphasize liquid handling (microfluidics, antifouling coatings), reference-electrode stability, and biochemical amplification for dissolved analytes. In hybrid platforms, combining VOC sensitivity (olfaction) with liquid specificity (taste) increases discriminability and lowers false positives when sampling and calibration are co-managed; mid-level fusion is commonly effective for such heterogeneous pairs [87].

Integrating these modality-specific advantages, such as volatile compound recognition ability from olfactory systems with high-throughput liquid-phase analysis from taste platforms, hybrid biosensor designs can achieve enhanced sensitivity, broader analyte coverage, and greater application versatility.

### 4.5. Challenges & Emerging Solutions

Despite significant progress have been made, several challenges hinder the translation of high-performance biomimetic olfactory and taste biosensors from laboratory prototypes to commercial products. Material degradation remains a major issue; natural biorecognition elements such as receptors, proteins, and organoid structures are susceptible to denaturation, chemical breakdown, and loss of activity under thermal or environmental stress. Even synthetic recognition elements, while tend to be more stable, can suffer from fouling, drift, and decreased sensitivity over time.

Scalability and reproducibility are additional hurdles. Complex immobilization processes, variations in nanomaterial synthesis, and batch-to-batch inconsistencies in biological components lead to fluctuations in performance. Achieving reliable mass production without sacrificing sensitivity or selectivity is especially important for regulated applications such as food safety and medical diagnostics.

Integration engineering components introduces additional challenges. Diverse materials, amplification schemes, and transducer platforms must be combined into compact, portable devices without sacrificing signal quality or durability. This is especially demanding for hybrid systems that combine olfactory and taste sensing, where cross-signal interference must be minimized.

Several practical solutions are emerging to address these issues. Self-healing and antifouling materials help preserve functionality during prolonged use. Modular, plug-and-play platforms allow for quick replacement of worn components. Environmentally friendly fabrication methods reduce cost and environmental impact. Advances in microfabrication and synthetic biology are producing more robust, miniaturized recognition elements, while machine-learning-guided calibration can adjust for drift and variability in real time. Together, these developments are paving the way for scalable, reproducible, and field-ready biosensing technologies.

## 5. AI & Data-Driven Enhancements

Data-driven techniques have evolved from being optional extras to becoming essential design tools for biomimetic olfactory and taste biosensors. Advanced machine learning (ML), deep learning, and neural-network models facilitate precise pattern recognition, real-time learning, and multimodal data fusion, thereby improving sensitivity, selectivity, and stability in varying conditions [3,24]. These approaches enable highly accurate detection of trace volatile or dissolved analytes and enhance signal processing, noise reduction, development of recognition elements, device calibration, and predictive analysis [95]. As a result, platforms used in -healthcare, environmental monitoring, food safety, and wearable technology are moving toward adaptive systems capable of self-updating.

**Figure 4:**
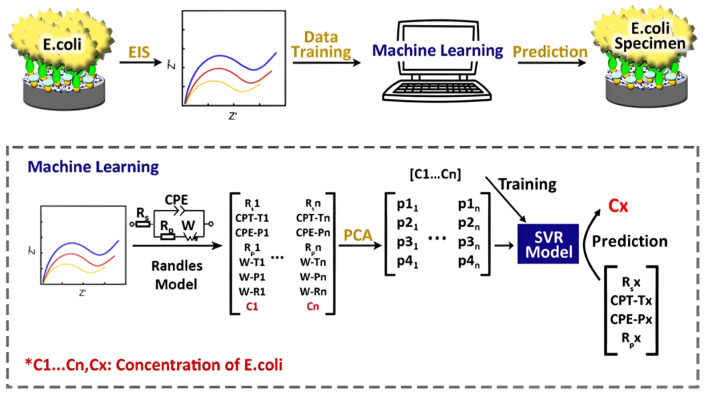
Schematic of an AI-driven biosensor workflow [96].

A representative workflow is shown in Figure 4, where raw electrochemical impedance spectroscopy (EIS) data are first parameterized using equivalent circuit modeling and then compressed through principal component analysis (PCA) to extract the most informative features. These reduced features are used to train a support vector regression (SVR) model that can accurately predict analyte concentrations in new samples. This pipeline exemplifies how AI and ML approaches convert complex, multidimensional biosensor outputs into actionable insights, demonstrating their value for real-time applications in food safety, medical diagnostics, and environmental monitoring [96].

### 5.1. Machine Learning for Detection

Machine learning (ML) has become fundamental for achieving accurate and reliable detection. Commonly used supervised algorithms, support vector machines (SVM), k-nearest neighbor (KNN), decision trees (DT), and artificial neural networks (ANN), which are widely used to classify volatile organic compound (VOC) patterns and taste profiles. Deep learning models such as convolutional (CNN) and recurrent neural networks (RNN) automatically extract features and often outperform traditional manual methods [25,97]. Beyond CNN/RNN, Transformer architectures are increasingly used for multi-channel sensor time series because self-attention captures long-range temporal dependencies and cross-channel interactions while handling variable sequence lengths. Variants such as Temporal Fusion Transformer, Informer, and PatchTST report strong results on real-world time-series benchmarks and provide interpretable attention maps valuable for feature attribution; lightweight or hybrid attention–convolution designs can lower compute for edge deployment [98]. Unsupervised techniques like principal component analysis (PCA) and clustering support exploratory data analysis, sensor drift identification, and dimensionality reduction, facilitating real-time deployment in portable devices [99].

An emerging strategy is theory-guided feature engineering, which combines domain knowledge from biosensing physics with ML processes to reduce training time, reduce overfitting, and improve explainability. For example, leveraging dynamic and transient response phases has been shown to minimize time-delay errors and false positives without sacrificing sensitivity [25]. Hybrid ML frameworks that integrate supervised, unsupervised, and deep learning components capitalize on the advantages of each method, improving classification accuracy across domains from food safety to clinical diagnostics [97].

Addressing sensor drift, a persistent challenge in long-term biosensing, remains a key focus. To address this, domain-adaptation models (e.g., DTSWKELM) transform cross-domain data so the classifier sees a steadier problem, reducing the need for fresh labeled data [100]. These strategies, particularly when combined with multi-source domain training, enhance robustness and extend device lifespans in practical settings. Drift is handled in layers: pre-processing/normalization (baseline correction, standardization) to suppress slow offsets and stabilize feature scales [101]; calibration transfer (DS/PDS/WPDS) to map drifted or new-device responses back into the source model space [93]; domain adaptation/transfer learning (e.g., DA-ELM, adversarial CDAN, multi-source attention) to align source–target feature distributions [102]; and online/incremental updates (OS-ELM/ODAELM) that refresh parameters with few target labels [103]. Together these steps keep decision boundaries stable as hardware ages [104].

Recent e-nose studies replace CNN/LSTM backbones with Transformer encoders and prototype-optimized unsupervised domain adaptation, improving average accuracy by ~11% to ~92.7% on public drift datasets—indicating strong potential for long-term robustness [105]. Generative adversarial networks can synthesize realistic spectra/voltammograms to balance classes and regularize training when data are scarce or imbalanced; GAN-based augmentation improves classification on imbalanced spectroscopy datasets, and VAE-GAN variants extract robust features from electrochemical-impedance series—approaches directly applicable to EIS-based e-tongues [106].

ML also plays a crucial role in sensor design optimization by guiding the selection of recognition elements, immobilization materials, and signal amplification strategies [46]. By predicting the most effective combinations of materials and architectures, ML accelerates prototyping and reduces experimental costs. Furthermore, coupling optical and electrical biosensing modalities with ML enables multimodal data fusion, combining their complementary strengths—optical systems offer label-free, high-sensitivity detection, while electrical platforms provide cost-effective, miniaturized formats.

Generative adversarial networks can synthesize realistic sensing signals (spectra/voltammograms or time-series) to balance classes and improve robustness when data are scarce or imbalanced; demonstrations include electronic-nose gas-sensor datasets and spectral sensing tasks [107]. Overall, the integration of advanced ML algorithms with biomimetic olfactory and taste biosensors represents a decisive step toward portable, reliable, and high-performance sensing technologies. These hybrid systems can meet demands in healthcare, environmental monitoring, food quality control, and defense, paving the way for autonomous, intelligent, and adaptive sensory devices [108].

### 5.2. Data Fusion Across Sensor Arrays

Integrating outputs from various olfactory and taste biosensors through data fusion has emerged as an effective approach to improve accuracy, stability, and adaptability in complex real-world settings. Fusion can be performed at three primary levels: low-level (merging raw data), mid-level (feature extraction and combination), and high-level (decision or prediction fusion) [87]. While low-level fusion is straightforward and preserves full signal detail, mid-level fusion often offers better performance in multi-variable datasets by reducing noise and retaining only the most relevant features. High-level fusion is particularly suited for classification tasks where independent models contribute complementary outputs.

Low-level fusion suits homogeneous arrays with matched sampling rates and SNR, preserving transients. Mid-level fusion is preferred for heterogeneous systems (e-nose + e-tongue), where feature selection or latent-space fusion reduces dimensional mismatch and noise. High-level fusion suits asynchronous modules or multi-site deployments, combining model outputs with confidence weighting; in all cases, synchronization and block scaling are prerequisites, and domain adaptation helps counter inter-module drift [87].

Similarly, combining electronic nose and tongue outputs with machine learning algorithms—such as SVM, PCA, or discriminant function analysis—has yielded remarkable classification accuracy, with some medical diagnostic applications achieving nearly perfect discrimination between disease conditions [109]. Beyond direct sensor pairings, fusion can extend to integrating complementary modalities such as multispectral imaging with e-nose data. Neuro-fuzzy regression models have effectively utilized this approach for tasks such as assessing meat freshness, even with limited dataset, by generating virtual samples to improve model robustness [110]. Advanced material-based enhancements, such as coupling nanomaterial-augmented biosensors with fusion algorithms, further boost sensitivity and selectivity in VOC detection and food quality monitoring.

Key challenges in cross-modal fusion include aligning datasets with disparate temporal dynamics, compensating for sensor drift, and managing heterogeneous noise sources. Nonetheless, emerging solutions—such as domain adaptation algorithms, multi-factor analysis (MFA), and AI-driven virtual sampling—are addressing these issues, paving the way for robust, multi-sensor platforms capable of high-confidence decision-making in practical applications.

### 5.3. Real-Time Analytics & IoT

The convergence of biomimetic olfactory and taste biosensors with the Internet of Things (IoT), edge computing, and cloud analytics is enabling real-time, scalable, and secure deployment across diverse application domains. By integrating biosensors with wireless communication technologies such as Bluetooth, Wi-Fi, and LoRaWAN, continuous and remote monitoring becomes feasible in areas such as healthcare diagnostics, food quality control, and environmental monitoring. Edge computing facilitates on-site data processing, which reduce latency and minimizing reliance on centralized systems, while cloud-based analytics pipelines support large-scale data storage, advanced pattern recognition, and predictive modelling [111,112].

On-device firmware performs baseline correction + feature extraction, and then a lightweight classifier/regressor (e.g., compact CNN/Transformer-lite or SVM) runs at the edge. A drift monitor (moving-window statistics) triggers calibration transfer or domain-adaptation jobs; encrypted features stream to the cloud for periodic retraining, fleet health, and audit logs, with OTA model updates back to devices. Sending features/gradients rather than raw signals helps preserve privacy while enabling continual learning [113].

Microfluidic and portable biosensing devices equipped with AI algorithms can perform on-device intelligent data filtering and classification before transmission, thus improving both privacy and response times [114]. Modular architectures combining edge intelligence for preliminary analysis and cloud intelligence for in-depth processing offer adaptability to varying application needs, from continuous patient monitoring to dynamic food supply chain assessment. In smart packaging, IoT-enabled biosensors can detect spoilage indicators in real time and relay the data to cloud platforms for proactive decision-making, enhancing traceability and reducing waste [115].

Despite these advances, security and scalability remain key challenges. Emerging solutions including pervasive AI for local data handling, blockchain integration for secure transaction records, and multi-hop IoT networks for robust connectivity. As 5G and next-generation wireless standards evolve, the synergy between AI-driven biosensing, edge computing, and IoT will likely expand their operational scope, creating resilient, adaptive systems capable of delivering reliable real-time sensory intelligence in complex settings.

### 5.4. Current Limitations

Despite rapid progress have been made, several limitations hinder the widespread deployment of AI-enhanced olfactory and taste biosensors. Technical challenges include a scarcity of high-quality, well-annotated datasets, variability in biosensor fabrication, and the short shelf-life of biological components, all of which can affect reproducibility and the ability of models to generalize. Computational constraints such as the black-box nature of deep learning models, high energy demands, and hardware limitations in portable systems, which hinder real-time applications. Data privacy and security concerns, particularly in cloud-connected and IoT-enabled platforms, remain unresolved, raising compliance issues under evolving regulatory frameworks. Moreover, AI models frequently have difficulty adjusting to new sensing conditions or shifts in data distribution, which diminishes their robustness in practical applications. Addressing these challenges will require advances in material stability, enhanced model interpretability, development of edge AI for energy-efficient inference, and the establishment of standardized protocols for secure data management [108,116,117].

## 6. Hybrid & Multiplexed Systems

Hybrid and multiplexed biosensing systems integrate olfactory and taste sensing modalities into a unified platform, enabling simultaneous detection of both volatile and non-volatile analytes. By combining complementary detection mechanisms, these systems overcome the limitations of single-modality approaches, significantly enhancing sensitivity, selectivity, and detection range. The synergy between modalities allows for richer chemical profiling, improved analyte discrimination, and resilience in complex sample matrices. Recent advances in materials science, signal processing, and AI-driven data fusion have accelerated their adoption in practical fields such as food quality control, medical diagnostics, environmental monitoring, and security screening. These platforms represent a next-generation paradigm for comprehensive, high-performance chemical sensing technologies [1,46].

### 6.1. Cross-Modal Olfactory–Taste Platforms

The integration of olfactory and taste biosensors into unified cross-modal platforms is inspired by the synergistic organization of human sensory systems, aiming to closely replicate multisensory perception. This approach combines complementary detection modalities—such as field-effect transistors, electrochemical sensors, and optical devices—into shared transduction layers that process both smell and taste signals. Core strategies include the incorporation of biorecognition molecules (e.g., membrane receptors and peptides) alongside advanced signal amplification methods using nanomaterials or enzymatic labels, which collectively enhance sensitivity, selectivity, and analyte discrimination [1].

Emerging designs such as the olfactory–taste synesthesia model (OTSM) process data from electronic nose (e-nose) and electronic tongue (e-tongue) systems in parallel, simulating neural integration pathways to improve classification accuracy and robustness in complex samples [118]. Neuromorphic architectures further enable super-additive responses by converting sensory inputs into spiking signals, enhancing detection performance under varying conditions [119].

These integrated platforms broaden the range of detectable analytes, reduce false positives, and enable richer sensory profiling in applications such as food quality assessment, environmental monitoring, and health diagnostics. By leveraging both biological inspiration and advanced computational models, cross-modal biosensors represent a promising step toward portable, intelligent systems capable of real-time, context-sensitive analysis in practical settings. Cross-modal sensing overcomes key single-modal limits (e.g., volatile-only or liquid-only blind spots, matrix/humidity interference, and look-alike mixture ambiguity that drives misclassifications/false positives) by fusing orthogonal volatilome and soluble/ionic cues, which consistently improves discrimination and robustness in complex food and biological samples [87].

### 6.2. Multifunctional Detection Architectures

Recent advances in multifunctional detection architectures have substantially enhanced the analytical performance and application scope of olfactory–taste hybrid biosensors. These architectures typically integrate diverse biorecognition molecules—such as membrane receptors, binding proteins, and engineered peptides—with tailored immobilization strategies and high-performance transducers, including field-effect transistors, electrode-based sensors, and fluorescence systems [1]. Signal amplification through nanomaterials, enzymatic labels, and nucleic acid techniques further boosts sensitivity and selectivity [46].

Microfluidic technologies provide precise control, miniaturization, and high-throughput sample handling, while portable smart devices extend detection capabilities to field and point-of-care settings. Integration with machine learning algorithms allows for advanced pattern recognition, improving analyte discrimination and robustness in complex environments. Novel approaches—such as biohybrid heterogeneous surfaces with tunable physicochemical properties—expand the chemical interaction spectrum, enabling fine discrimination among volatile and dissolved analytes [120].

By combining biomimetic design, advanced materials, microfluidics, and intelligent data processing, these architectures deliver synergistic improvements in sensitivity, specificity, and portability. Such multifunctional systems show great potential for diverse applications, including food quality monitoring, environmental monitoring, and medical diagnostics, representing a critical step toward fully integrated, real-world-ready olfactory–taste biosensing platforms.

### 6.3. Application Scenarios in Food, Environment, and Healthcare

Hybrid olfactory–taste biosensors consistently outperform single-modality systems in applications requiring ultra-high sensitivity, specificity, and multiple-analyte discrimination. In food safety and quality control, these systems enable rapid and precise detection of spoilage indicators, biogenic amines, and foodborne pathogens, achieving detection limits as low as the femtomolar level and near-perfect classification accuracy within seconds [121]. The synergy of olfactory and gustatory sensing, coupled with advanced materials such as graphene, nanohybrids, and hierarchical nanostructures, enhances signal amplification, stability, and selectivity [46]

In biomedical diagnostics, these hybrid platforms improve the detection of diverse biomarkers—including metabolites, neurotransmitters, and cancer cells—facilitating early diagnosis and personalized healthcare. Environmental monitoring and security applications also benefit from heightened analyte discrimination in complex matrices, enabling reliable detection of volatile explosives and environmental toxins.

**Figure 5:**
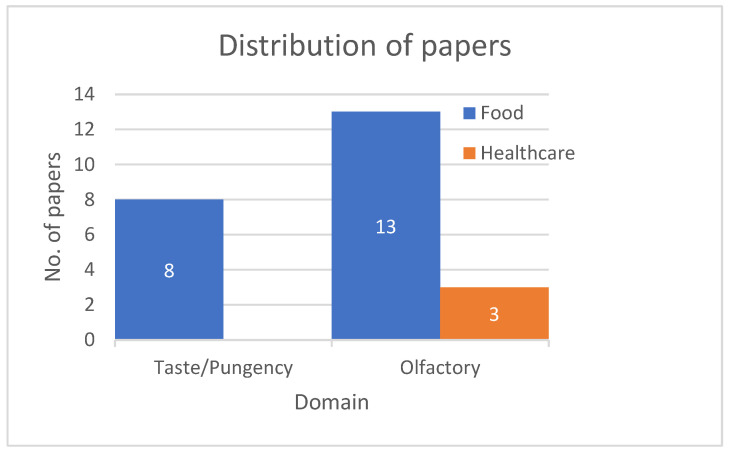
Distribution of included papers across taste and olfactory biosensors domain.

The integration of microfluidics, bioelectronic devices, and machine learning further refines performance by enabling real-time analysis, reduced sample volumes, ensuring reliable operation in field conditions. Collectively, these improvements mark hybrid olfactory–taste biosensors as transformative tools for multidisciplinary sensing challenges. Distribution of included use cases across taste and olfactory in Table 3 and Table 4 is shown in the barchart given in Figure 5.

## 7. Pathways to Market Readiness

### 7.1. Current Market Landscape

The commercial landscape for olfactory and taste biosensors is steadily expanding, driven by technological advancements in AI integration, device miniaturization, and IoT connectivity. These trends are transforming biosensors from specialized research tools into scalable products for industries such as food safety, environmental monitoring, security, and healthcare [24]

Several companies are emerging as leaders in this field. Aryballe Corporation has developed portable devices for odor detection with applications ranging from the automotive industry to food quality monitoring. Koniku Corporation integrates machine learning with biosensor platforms for automated odorant identification, targeting both security and health diagnostics. Intel Corporation is also contributing AI-driven architectures to enhance biosensor performance and adaptability [1].

A recent market analysis identified 44 companies and 265 commercial electronic nose (e-nose) products, classified by sensing mechanism: chemiresistors (12.8%), electrochemical sensors (13.0%), catalytic beads (12.4%), and optical detection techniques (16.0%) (IR/NDIR/FTIR (14.6%) + UV/UV-IR (1.4%), non-spectroscopic optical modalities (7.1%), GC/MS-based devices (10.8%), photoionization/flame-ionization detectors (PID/FID; 8.2%), surface acoustic wave (SAW; 2.5%), quartz crystal microbalance (QCM; 0.2%), ion mobility spectrometry (IMS; 3.7%), and “others” categories (13.3%). The dominant applications are in industrial and chemical/petrochemical fields [122]. Although food and beverage applications currently account for ~6% of the market share, this segment is growing rapidly due to increasing concerns about safety, sustainability, and quality control.

Specialized markets, such as the wine industry, are adopting e-nose technologies for tasks like quality grading, defect detection, and geographic origin authentication, with future improvements expected from AI-driven volatile compound analysis [123]. These developments suggest that as biosensor technologies continue to advance and become more cost-effective, their adoption will accelerate across a variety of industries.

### 7.2. Technical Challenges

The widespread adoption of biomimetic olfactory and gustatory biosensors is hindered by multiple technical, environmental, and operational challenges. A major challenge is maintaining stability and reproducibility, since biological components such as olfactory receptors and other biorecognition elements tend to degrade over time, leading to sensor drift and lowering accuracy in detecting trace-level gaseous or dissolved analytes [1,124]. This instability is exacerbated in long-term deployments, where calibration drift becomes significant, especially under fluctuating environmental conditions like temperature, humidity, and airborne contaminants [3].

Environmental robustness is another critical limitation. Harsh or variable real-world conditions can reduce sensitivity, specificity, and durability, affecting detection limits and repeatability. Moreover, sample complexity—especially in real-world scenarios involving mixtures of volatile and non-volatile compounds—can introduce nonspecific binding and signal interference, challenging the accuracy of biomarker identification [2].

From a materials and engineering perspective, immobilization of sensitive recognition elements onto transducers remains difficult. Achieving uniform distribution, strong binding, and biocompatibility is essential to maintain both signal integrity and long-term stability [1]. While nanomaterials, metal–organic frameworks, and genetically engineered receptors offer potential performance gains, issues like impurity control, poor conductivity, and electrical instability hinder practical application [46].

Addressing these barriers requires multidisciplinary strategies: advanced immobilization materials (e.g., extracellular matrices and synthetic hydrogels), robust calibration algorithms, environmental shielding, and nanotechnology-based enhancements to improve sensitivity and selectivity. Continued innovation in materials science, microfluidics, and machine learning integration will be pivotal to overcoming current limitations and enabling reliable, scalable biosensor deployment.

### 7.3. Regulatory and Manufacturing Barriers

The commercialization of olfactory and taste biosensors faces multiple regulatory and manufacturing hurdles that directly affect their scalability and market penetration. Ensuring reliability and standardization remain remains a fundamental issue, as these biosensors must meet stringent international standards such as those from the FDA, ISO, CE marking, and, for AI-driven systems, the emerging EU AI Act [3]. These frameworks require validated performance metrics, traceability, and consistent sensor operation, yet differences in global regulatory pathways further complicate compliance.

From a manufacturing perspective, scalability, reproducibility, cost, and quality assurance are critical constraints. Variations in biosensor performance across production batches, fragility of biorecognition elements, and the complexity of detection methods limit high-volume manufacturing [46]. Ensuring reproducibility while keeping costs competitive requires advances in synthetic biology, microfabrication, and stable immobilization techniques.

Regulatory challenges also overlap with concerns about safety, toxicity, and environmental sustainability. To address these issues, strategies such as developing environmentally friendly (“green”)biosensors, integrating AI and ML into manufacturing for predictive quality control, and fostering collaborative standard-setting among researchers, industries, and policymakers have been proposed [117]. Nanotechnology offers additional promise in overcoming performance and reproducibility issues, particularly in odor localization and sensor stability, but requires alignment with safety and compliance frameworks [2].

Overall, bridging these gaps will require harmonized international standards, robust quality management systems, and manufacturing innovations that ensure scalability without sacrificing accuracy, stability, or environmental responsibility. Bringing the algorithmic pieces together, Table 5 surveys AI workflows deployed, spanning classical PCA/SVM pipelines, domain-adaptation for sensor drift, temporal deep learning models, and mid- to high-level data fusion. It highlights typical improvements in accuracy, robustness, and deployment scope (edge vs. cloud), while also identifying unresolved issues such as dataset quality, persistent drift, interpretability, and reporting deficiencies. This provides a concise checklist to support reproducible benchmarking and practical deployment.

### 7.4. Pathways to Market

Accelerating the market entry of olfactory and taste biosensors requires a coordinated approach that integrates design, engineering, and commercialization strategies. Design for manufacturability (DFM) emphasizes miniaturization and intelligent system integration to produce compact, portable devices with robust detection performance. Advances in microfabrication, synthetic biology, and nanotechnology enable manufacturable solutions while maintaining high sensitivity and stability [1,46]

Cost reduction can be achieved through scalable production techniques, microfluidic integration, and the use of affordable nanomaterials, ensuring competitive pricing without sacrificing analytical performance [2]. AI-driven design can further reduce costs by optimizing material properties and detection workflows [24].

A user-centric interface is critical for adoption, with emphasis on intuitive operation, clear data visualization, and compatibility with smartphones, cloud services, and IoT networks for real-time analysis and remote monitoring. AI/IoT integration also enhances autonomous decision-making and predictive maintenance capabilities.

Business model innovation should align with target markets, from direct-to-consumer health monitoring devices to B2B solutions in food safety, environmental monitoring, and medical diagnostics. Subscription-based data services, industry-specific licensing, and value-added analytics packages can expand revenue streams [1,24].

Finally, strategic partnerships with industry leaders, healthcare providers, and regulatory agencies can facilitate scale-up, regulatory approval, and global distribution. Collaborations with technology firms, such as AI and semiconductor companies, can accelerate product refinement and market readiness [2]. By integrating these pathways, the commercialization of hybrid biosensors can transition from prototype to scalable, market-ready solutions, unlocking their potential in diverse real-world applications.

## 8. Future Directions

The rapid progress in biomimetic olfactory and taste biosensors demonstrates both the promise and the challenges of creating reliable, scalable, and intelligent sensing platforms. Looking ahead, several key directions emerge from current research that can guide future progress and practical application.

### 8.1. Multidisciplinary Integration

Future progress will depend on tighter integration across various fields. Advances in receptor engineering, nanomaterials, microfluidics, and artificial intelligence have so far propelled the field independently, but the greatest breakthroughs will arise from integrated design approaches that combine chemistry, device engineering, and computational intelligence [1,46]. Such integration will improve stability, reproducibility, and robustness under real-world conditions, where isolated innovations often fall short.

### 8.2. Adaptive and Self-Learning Biosensors

A key challenge is sensor drift caused by environmental factors, material degradation, or bio-interface instability. To overcome this, future biosensors must incorporate adaptive learning frameworks capable of recalibration in situ. Machine learning methods, including domain adaptation and federated learning, can support continuous operation across different settings while maintaining interpretability and privacy [24]. Embedding such models at the edge will further reduce latency and dependence on centralized data infrastructures.

### 8.3. Hybrid and Cross-Modal Architectures

Olfactory systems are excellent at detecting volatile fingerprints, whereas taste sensors provide robust quantitation in liquid phases. Hybrid, cross-modal biosensors that integrate both modalities will remain central to broadening chemical coverage and reducing false positives [1,145]. Multifunctional detection architectures that combine nanomaterials, organoid models, and microfluidics are expected to enhance sensitivity and selectivity, enabling applications in food safety, environmental monitoring, and medical diagnostics [146].

### 8.4. Sustainability, Portability, and Manufacturability

For large-scale adoption, future devices must balance high performance with sustainability and accessibility. Research should focus on developing portable, low-power systems that integrate with IoT networks for real-time monitoring. Employing cost-effective, environmentally friendly materials and optimizing manufacturability will be essential for global deployment, especially in resource-limited areas [115,147]. Green sensor technologies, supported by AI-driven design, will also reduce ecological and economic barriers [117].

### 8.5. Open Datasets and Collaborative AI

A recurring limitation is the scarcity of high-quality datasets for training robust AI models. Establishing open, standardized datasets that capture diverse analytes, environmental contexts, and long-term drift effects will be critical for advancing machine learning in biosensing [108]. Collaborative consortia across academia, industry, and regulators can facilitate benchmarking, reproducibility, and shared validation protocols, helping to avoid fragmented progress and accelerating technology readiness.

### 8.6. Regulatory Alignment and Market Translation

Commercialization will require harmonized regulatory frameworks that address performance, safety, and ethical use. Emerging efforts such as the EU AI Act and WHO-led guidelines may offer models for biosensor regulation [116]. To accelerate adoption, early engagement with regulators, end-users, and industry stakeholders will be essential for aligning claims, risk controls, and usability. Business model innovation—particularly partnerships with healthcare, food, and environmental industries—will provide practical pathways for scaling deployment [24,122].

## 9. Conclusions

Biomimetic olfactory and taste biosensors have evolved from conceptual prototypes into versatile analytical tools, empowered by advances in receptor engineering, nanomaterials, microfluidics, and AI-driven signal processing. Machine learning now underpins critical functions—drift correction, feature recognition, and multi-sensor data fusion—enabling reliable detection of complex mixtures at trace levels in real-world environments. Progress increasingly depends on co-design: aligning chemistry, interfaces, devices, and algorithms rather than optimizing each in isolation. Comparative insights confirm that olfactory sensors are particularly effective for detecting volatile compounds, while taste sensors provide robust quantification of dissolved analytes; in addition, hybrid platforms combine these strengths to expand chemical coverage, lower error rates, and enhance resilience to noise. When integrated with portable electronics and microfluidics, such systems are well-positioned for applications in food safety, medical diagnostics, environmental monitoring, and security.

The next phase requires turning technical promise into scalable, trustworthy solutions. Rather than restating known hurdles, the focus must be on actionable pathways: establishing open benchmarks for long-term stability, developing modular recognition layers to ease replacement and upgrades, deploying interpretable AI at the edge for adaptive calibration, and aligning manufacturing processes with regulatory expectations from the outset. Strategic engagement with industry and regulators will be essential for harmonized standards and accelerated adoption. By advancing along these coordinated pathways, hybrid smell–taste biosensing can evolve into a dependable layer of chemical intelligence across healthcare, food systems, urban infrastructure, and environmental monitoring.

## Figures and Tables

**Table 1:** Comparison between biomimetic smell/taste biosensors and commonly used non-biological sensors.

Aspect	Biomimetic Smell/Taste Biosensors	MOX/MQ Chemiresistors
Operating temp	Room temperature (hydrated biointerfaces; aqueous-compatible for taste) [27]	Typically 200–400 °C; heaters/micro-hotplates required [28].
Selectivity	Molecular recognition → high specificity; multiplexable panels of OR/OBP [27].	Cross-sensitive; selectivity via arrays + ML [29].
Typical LOD	fM–pM (e.g., OR-CNT-FET 1 fM geraniol; reports down to 0.04 fM) [30].	ppb–ppm typical (low-ppb possible in lab) [31].
Response/recovery	Seconds–minutes (binding/transport limited) [27].	Seconds response; recovery often tens of seconds and condition-dependent [32].
Humidity effects/drift	Bio-layers need hydration; stability depends on immobilization [33].	Humidity strongly interferes; pronounced short/long-term drift [34].
Power	Low (no heater) [35].	Tens of mW typical (e.g., ~60 mW @ 400 °C; ~20 mW in SiC design) [36].
Maintenance	Bio-layer shelf-life/rehydration; OBPs thermally robust (~70–75 °C) [37].	Periodic calibration; contamination/heater aging [32].
Sample compatibility	Works in aqueous/complex matrices (taste) and gas (olfaction) with interfaces [35].	Optimized for gas-phase VOCs/inorganics at elevated T [38].
Multiplexing	Natural panelization with multiple ORs/OBPs [33].	Arrays are standard in e-nose systems [29].
Warm-up	Minimal (no heater) [35].	Thermal stabilization needed [38].

**Table 2:** Publisher domains of the cited articles.

Domain	Count	Percentage
MDPI	56	38.10%
Elsevier/ScienceDirect	38	25.85%
ACS Publications	13	8.84%
Wiley Online Library	9	6.12%
Nature/Springer Nature (nature.com)	7	4.76%
SpringerOpen/BMC (non-nature.com)	3	2.04%
Frontiers	4	2.72%
RSC Publishing	2	1.36%
IEEE Xplore	2	1.36%
ECS Digital Library	2	1.36%
AAAS (Science Advances)	2	1.36%
Others	9	6.12%
Total	147	100%

**Table 3:** Biomimetic taste/pungency biosensors.

Recognition Element & Sensitive Material	Transducer Type	Target Analyte(s)	Detection Limit/Range	Selectivity & Binding/Energy Constants	Application	References
Venus flytrap domain (T1R2) on CNTs	CNT-FET	Sucrose & saccharin	0.1 fM; 0.1 fM–1 μM	Selectivity for cyclamate, tasteless disaccharide, l-monosodium glutamate & denatonium; Kd ≈ 2.05 × 10^−11^, 6.88 × 10^−12^ M	Sweetener detection in food/beverage quality control	[59]
Nanovesicles containing AmGr10	Nanovesicle-FET	l-Monosodium glutamate	100 pM; 100 pM–10 μM	Selective versus sucrose & phenylthiocarbamide; Kd ≈ 1.77 × 10^8^ M^−1^	Liquid food analysis	[60]
Nanovesicles containing rTRPV1	CNT-FET	Capsaicin, allicin, sanshool	LOD ≈ 1 pM; responses demonstrated up to µM level	Selective to TRPV1 agonists; negligible response to non-pungent tastants	Pungency evaluation, food screening	[61]
hTAS2R46 nanodiscs	CNT-FET	Denatonium benzoate	LOD ≈ 0.1 nM; range 0.1 nM–1 µM	Specific to denatonium; minimal cross-response; Kd/Ka: Equilibrium constant = 0.1354 pM^−1^, i.e., Kd ≈ 7.4 nM	Bitterant detection	[62]
hT1R1 immobilized on a glassy carbon electrode (GCE)	Electrochemical (signal amplification on GCE)	Sodium glutamate, disodium inosinate, disodium guanylate, disodium succinate	Detection limits: 1.5 pM, 0.86 pM, 2.3 pM & 0.86 pM; detection range 10^−14^–10^−12^ M	Association constants Ka ≈ 7.42 × 10^−16^–2.25 × 10^−15^ M	Food taste analysis	[63]
Venus flytrap domain of T1R1 on an electrode	Electrochemical (differential pulse voltammetry	Inosine-5′-monophosphate, monosodium L-glutamate, beefy meaty peptide, sodium succinate	Detection limit ~0.1 pM; ranges 10^−13^–10^−6^ M (IMP), 10^−13^–10^−8^ M (MSG) and 10^−13^–10^−7^ M (BMP)	RSD 2.3–3.2%	Food taste analysis	[64]
TRPV1 cell membrane on an electrode	Electrochemical (impedance-based)	Capsaicin, allicin, sanshool	Detection limits 1 × 10^−15^ M, 1 × 10^−14^ M & 1 × 10^−15^ M; detection ranges up to 10^−12^ M	Association constants Ka ≈ 3.52 × 10^−16^–5.02 × 10^−15^ M	Food additive analysis	[65]
Odorant-binding protein-based biosensor	Electrochemical impedance spectroscopy (EIS)	Bitter taste molecules	Linear response 10^−9^–10^−6^ mg/mL	OBP selectivity for bitter ligands	Bitter taste detection	[66]

**Table 4:** Biomimetic olfactory biosensors.

Recognition Element & Sensitive Material	Transducer Type	Target Analyte(s)	Detection Limit/Range	Selectivity & Binding/Energy Constants	Application	References
OR2J2, OR2W1, TAAR5 & TAS2R38 on multi-channel CNTs	Multi-channel swCNT-FET	Octanol, hexanol, trimethylamine, goitrin	pM (100 fM–100 nM)	Kd ≈ 7.53 × 10^12^–1.78 × 10^12^ M^−1^ for different receptors	Food safety	[67]
hOR2AG1 & hOR3A1 on graphene-mediated FET	GMs-FET	Amyl butyrate & helional	0.1 fM; 0.1 fM–10 pM	Selective against butyl & pentyl butyrate; piperonal & safrole; Kd ≈ 2.93 × 10^15^ & 9.6 × 10^14^ M^−1^	Spice industry	[68]
Micelle-stabilized ODR-10 receptor	Electrolyte–insulator–semiconductor FET	Diacetyl	10 fM; 1 fM–10 nM	Selective versus 2-butanone & 3-methyl-2-butanone; K ≈ 1.7 × 10^−12^ M	Alcoholic beverages	[69]
OBP-derived peptide on CNT-FET	CNT-FET	3-Methyl-1-butanol	1 fM; 1 fM–10 nM	Interferents: 2-methylbutane, methyl isopropyl ketone, 3-methyl-1-butanethiol, isobutyl acetate, 3-methylbutanal & 3-methylbutanoic acid; Kd ≈ 5.25 × 10^13^ M^−1^	Pathogen-contaminated food	[1]
Mutant porcine OBP (pOBP-F88W)	Water-gated OFET	Carvone enantiomers	LOD = 50 pM; LQ = 150 pM	Enantioselectivity factor ≈ 6.3; Kd = 0.81 nM (S), 20 nM (R)	Chiral flavour QC	[35]
hOR1A2 nanodiscs on CNT-FET	CNT-FET	Geraniol, citronellol	LOD = 1 fM (geraniol), 10 fM (citronellol); range up to 1 µM	Specific binding; discriminated rose odorants from other compounds	Fragrance analysis	[70]
TAAR13c/d nanodiscs	Side-gated graphene FET	Cadaverine, putrescine	LOD = 1 fM; range 1 fM–10 pM	Highly specific; stable under low humidity	Fish spoilage monitoring	[71]
OBP14 from Apis mellifera	rGO-FET	Homovanillic acid (HVA), methyl vanillate, eugenol	LOD ≈ 100 nM; range 100 nM–3.3 mM	Binding order HVA > methyl vanillate > eugenol; Kd range ≈ 4 μM to 3.3 mM	Cosmetic/fragrance detection	[72]
ORP (olfactory receptor-derived peptide)	Flexible SWCNT sensor	Trimethylamine (TMA)	LOD = 0.1 ppq; broad dynamic range	High selectivity to TMA; effective even in seafood matrices	Real-time fish spoilage detection	[73]
Water-gated OFET with porcine OBP	WGOFET	S(+)/R(−)-carvone	LOD = 1 pM; range 1 pM–5 nM	Enantioselectivity factor ≈ 6.3; K ≈ 0.81 nM	Mint flavour QC	[74]
OBP-derived peptide (ORP) used	Single-walled CNTs (SWCNTs) on silicon pyramid structure	Trimethylamine (TMA) vapor	LOD ≈ As low as 0.01 parts per trillion (ppt)	Highly selective vs. other amines; stable >30 days	Fish spoilage detection	[75]
Peptide receptor derived from olfactory receptor	Bioelectronic nose (CNT-FET)	Trimethylamine (TMA)	LOD = 10 fM	Selective detection in mixed seafood vapors	Seafood freshness quality control	[76]
ORP (olfactory peptide) + microfluidic system	Microfluidic-integrated bioelectronic nose	Trimethylamine (TMA)	LOD = 10 ppt	Good selectivity in vapor phase; reusable	Portable gas-phase seafood monitoring	[77]
ORP immobilized using Steglich esterification + NCL	Modified CNT-FET	Trimethylamine (TMA)	LOD = 0.01 ppt	Improved binding efficiency; high specificity	Environmental and food odor sensing	[78]
Gold disk electrodes with liposomes containing Or10a, Or22a & Or71a	Electrochemical impedance spectroscopy (EIS)	Methyl salicylate, methyl hexanoate, 4-ethylguaiacol	Detection limits 1 pM, 1 fM & 0.1 fM; detection ranges 10^−13^–10^−7^ M, 10^−15^–10^−7^ M & 10^−17^–10^−9^ M	–	Cosmetics & medicine	[79]
Olfactory receptor OR7D4 immobilized via His-tag on gold electrode	Amperometric (SWV)	Androstenone (boar taint compound)	Linear response 10^−14^–10^−4^ M	OR specificity for androstenone	Meat quality control	[80]

**Table 5:** AI and data-driven enhancements in biosensing.

Study/System	Data & Sensors	Machine Learning Method	Performance Metrics	Application	References
Artificial olfactory system based on human ORs	Conductance patterns from human OR nanodiscs on synaptic devices; single & mixed short-chain fatty acids (SCFAs) at 3 ppm	Principal component analysis (PC1 + PC2 explained 97.9% variance) followed by custom artificial neural network (27 input –14 hidden –4 output neurons)	Detection limits 0.07–1.30 ppm; odor recognition accuracy reached 100% after 100 training steps (epochs) and remained 90–100% after 1 000 epochs; mixed-odorant recognition accuracy reached 91.6%	Molecular odorant discrimination & neuromorphic sensing	[125]
Data fusion of electronic nose and electronic tongue for prostate cancer detection	Breath and urine samples analyzed by gas sensor arrays (e Nose) and electrochemical electrodes (e Tongue)	Data fusion with multivariate classification algorithms (e.g., support vector machines, principal component analysis, discriminant function analysis)	Combining sensory data yielded 100% classification accuracy for distinguishing prostate cancer, benign prostate hyperplasia, prostatitis and controls; e Tongue alone achieved 92.9% accuracy	Non-invasive prostate cancer diagnosis	[126]
Neuromorphic artificial gustation with layered GO membranes	Layered graphene oxide (GO) membranes operating in liquid; generates ionic current patterns in response to basic tastes (sweet, sour, salty, bitter) and complex beverages (Coke, coffee, wine, etc.)	Reservoir computing: on-membrane ionic dynamics act as physical reservoir; processed by external neural network classifier	~98.5% accuracy for known basic tastes; 75–90% accuracy for novel taste inputs; ~96% for complex beverages; on-membrane memory retention up to ~140 s	Artificial tongue for beverage classification, taste restoration, health and authenticity monitoring	[127]
Adaptive ML for forensic e-nose (VOC scent signatures)	Portable e-nose arrays measuring biosamples (breath/tissue volatiles)	Adaptive machine learning pipeline with transfer learning	98.1% (postmortem vs. antemortem), 97.2% (human vs. animal); high-resolution PMI estimation	Forensic scent detection	[128]
Prototype-Optimized UDA for drifted e-nose	MO_x_ e-nose data with temporal drift	Unsupervised domain adaptation using dynamic Transformer encoder + prototype learning	Outperforms prior UDA baselines; average accuracy improved by ~11%, reaching 92.67% on CQU dataset	Sensor drift compensation in e-nose	[105]
Semi-supervised deep learning for gas-sensor drift	Resistive gas sensor arrays	Semi-supervised domain-adaptive CNN using ensemble classifiers, multi-level features, MMD-based pretraining loss, and center loss	76.06% accuracy (long-drift), 82.07% accuracy (short-drift), R^2^ = 0.804 in regression, outperforming conventional methods	Drift compensation in e-nose systems	[129]
Cross-domain active learning for e-nose drift	Electronic nose datasets collected across time to reflect domain drift (sensor aging)	Cross-Domain Active Learning (CDAL): Active Learning (Hellinger Distance) + Domain Adaptation (MMD), combined via weighted sample selection	~10% higher average accuracy than baselines; short-term drift accuracy > 82% with ~30 labeled samples	Drift compensation in e-nose systems	[130]
In-sensor reservoir computing olfactory neuron	Bionic olfactory neuron device: OFET array providing in-sensor reservoir dynamics	Physical reservoir computing (RC) + KNN classifier	100% accuracy for 8 gases; 99.04% accuracy for 26 gases (including mixtures, isomers, homologs)	Edge olfaction/neuromorphic sensing	[22]
Cascadable OECT for multimodal edge sensing	cv-OECT (vertical-traverse organic electrochemical transistor) acting as a multimodal sensor (ions, light, temperature, ECG, taste)	In-hardware neural network for edge computing via reservoir computing, SNN/ANN with STDP	Demonstrated multi-modal sensing capabilities; 10-bit analog memory with >10,000 s retention; simulated 100% classification accuracy for ECG/MNIST datasets	On-device AI for biosensing/neuromorphic sensing	[131]
E-nose + E-tongue fusion for prostate cancer	Breath e-nose and urine e-tongue (C110/250BT electrodes)	PCA/DFA/SVM/SVM-KNN/Random Forest fusion techniques	Fusion: 100% accuracy; E-tongue alone: 92.9% accuracy	Non-invasive prostate cancer diagnosis	[109]
E-nose for prostate cancer in urine (clinical)	Urinary VOCs via e-nose	Pattern recognition using sensitivity, specificity, AUC (no ML specified)	Sensitivity: 85.2%; Specificity: 79.1%; AUC: 0.821	Non-invasive prostate cancer diagnosis	[132]
Neural-network e-nose for prostate cancer (urine)	MOOSY-32 e-nose analyzing urinary VOCs	Feedforward neural network with redundancy strategy	Recall (sensitivity) of 91% for detecting prostate cancer	Rapid, non-invasive point-of-care prostate cancer detection	[133]
Breath e-nose for colorectal cancer screening	Exhaled breath VOCs via e-nose	Supervised classification (e.g., SVM, RF)	Overall: AUC 0.87, sensitivity 0.81, specificity 0.85; Early-stage: sens 0.90, spec 0.85; Non-smokers: sens 0.88, spec 0.92	CRC complementary screening tool (adjunct to FIT)	[134]
E-tongue impedance + ML for oral cancer (saliva)	Microfluidic impedance e-tongue analyzing saliva	Supervised classifiers (SVM-RBF, Random Forest, etc.)	>80% accuracy (binary classification), ~70% for multi-class; improved with clinical data	Non-invasive oral cancer diagnostics	[135]
E-tongue for wastewater Pb detection	Electronic tongue analyzing coal mining wastewater VOC/signals	Supervised classifiers (SVM, Random Forest, k-NN, Naïve Bayes, QDA)	Accuracy ~90%; Precision ~90–91%; AUC ~94–94.5%; high sensitivity/specificity and F_1_-score all similarly high	Environmental monitoring (lead detection in wastewater)	[136]
Polypyrrole smart e-tongue for coffee quality	Polypyrrole-based voltammetric sensor array (7 doped electrodes)	PCNN (PCA + neural networks) & cluster analysis	Accurate discrimination of five coffee varieties via unique fingerprints	Coffee quality/authenticity detection	[137]
Voltammetric e-tongue + custom preprocessing	Voltammetric e-tongue analyzing tomato purée signals	Custom preprocessing + LDA classifier	Average F_1_-score: 99.26% (across 100 runs)	Rapid tomato cultivar discrimination	[138]
Learning-efficient deep models for e-nose datasets	MOx sensor wind-tunnel dataset (public) + custom dataset in hood setting	Comparison of deep models (DNN, CNN, LSTM) vs. boosting models	Boosting models showed faster learning and higher robustness/accuracy	Algorithm benchmarking for e-nose signals	[139]
Low-cost e-nose for urinary infections	Urine headspace detected by portable e-nose	PCA; SVM classifier	PCA: 49% accuracy; SVM: 74% accuracy	Point-of-care UTI detection	[140]
Data fusion review for food/drink authentication	E-nose, e-tongue, imaging (electronic eye)	Overview of low-, mid-, high-level fusion strategies	Improved classification accuracy versus single modality	Food/drink authentication	[141]
E-nose advances for VOC breath diagnosis	Exhaled-VOC e-nose platforms	Pattern recognition & deep learning (review)	High sensitivity & rapid response (as reviewed)	Disease diagnostics via breath analysis	[142]
Systematic review of e-nose/e-tongue for contaminants	Multiple sensor modalities (e-nose, e-tongue)	Survey of ML/chemometric methods (PCA, LDA, PLS-DA, SVM, ANN, etc.)	High sensitivities reported in case studies (e.g., 100% for soft-rot); overall aggregate accuracies (like 96.83%, 94%) are not explicitly reported	Food contaminant detection	[143]
Reservoir computing overview for edge AI sensing	Physical reservoir devices (e.g., memristive, ionic/optoelectronic)	Survey of physical reservoir computing	Emphasized design for low-latency, energy-efficient inference	Edge biosensing & multimodal fusion	[144]

## Data Availability

No new data were created or analyzed in this study.

## Abbreviations

The following abbreviations are used in this manuscript:

ANN	Artificial Neural Networks
AI	Artificial Intelligence
CNN	Convolutional Neural Networks
CNT	Carbon Nanotube
DFM	Design for Manufacturability
DT	Decision Trees
e-Nose	Electronic Nose
e-Tongue	Electronic Tongue
EIS	Electrochemical Impedance Spectroscopy
FETs	Field-Effect Transistors
GCE	Glassy Carbon Electrode
GPCR	G Protein-Coupled Receptor
ISFETs	Ion-Sensitive FETs
IoT	Internet of Things
KNN	k-Nearest Neighbor
LOD	Limit of Detection
MEA	Microelectrode Array
MEA	Multi-Factor Analysis
MIPs	Molecularly Imprinted Polymers
ML	Machine Learning
MOFs	Metal–Organic Frameworks
OECTs	Organic Electrochemical Transistors
OFET	Organic Field-Effect Transistor
OSNs	Olfactory Sensory Neurons
OR	Odorant Receptor
OTSM	Olfactory–Taste Synesthesia Model
PCA	Principal Component Analysis
QCM	Quartz Crystal Microbalance
RNN	Recurrent Neural Networks
SPR	Surface Plasmon Resonance
SVM	Support Vector Machines
SVR	Support Vector Regression
TMDCs	Transition Metal Dichalcogenides
VOC	Volatile Organic Compound
